# From Intrinsic Resin Properties to Interlaminar Fracture Toughness of CFRP: Crack-Tip Deformation, Transfer Mechanisms, and Loading-Mode Dependence

**DOI:** 10.3390/polym18111366

**Published:** 2026-05-31

**Authors:** Xiuxiang Li, Yunfu Ou, Juan Li, Yiting Weng, Yunxiao Zhang, Anran Fu, Xia Liu, Qizhong Huang, Dongsheng Mao

**Affiliations:** 1 School of Materials Science and Chemical Engineering, Ningbo University, Ningbo 315211, China; 2 State Key Laboratory of Advanced Marine Materials, Ningbo Institute of Materials Technology and Engineering, Chinese Academy of Sciences, Ningbo 315201, China; ouyunfu@nimte.ac.cn (Y.O.);; 3 School of Materials Science and Engineering, NingboTech University, Ningbo 315100, China; 4 College of Materials Science and Opto-Electronic Technology, University of Chinese Academy of Sciences, Beijing 100049, China; 5 School of Chemical Sciences, University of Chinese Academy of Sciences, Beijing 100049, China; 6 Naval Medical Center, Naval Medical University, Shanghai 200433, China

**Keywords:** CFRP, ILFT, fracture mechanism, resin matrix, crack propagation

## Abstract

Interlaminar fracture toughness (ILFT) is a key factor governing the damage tolerance and service reliability of carbon fiber-reinforced polymer (CFRP) laminates. This study aims to clarify how the deformation capability of epoxy resin affects the Mode I and Mode II ILFT of carbon fiber/epoxy laminates under comparable fiber, resin-content, and laminate-configuration conditions. Two epoxy systems were compared: a high-strength/high-modulus (HSHM) resin system, designated as Group B, and a high-toughness (HT) resin system, designated as Group T. Neat resin castings were characterized by tensile and flexural tests, and the corresponding CFRP laminates were evaluated using double cantilever beam (DCB) and end-notched flexure (ENF) tests. Although Group T showed slightly lower tensile strength and modulus than Group B, its elongation at break increased from 4.0% to 6.5%, corresponding to an increase of approximately 62.5%. The Mode I ILFT (GIC) increased from approximately 279 J/m^2^ for Group B to 487 J/m^2^ for Group T, while the Mode II ILFT (GIIC) increased from approximately 530 J/m^2^ to 708 J/m^2^, corresponding to improvements of approximately 74.6% and 33.6%, respectively. Scanning electron microscopy (SEM) observations indicated that Group T promoted more resin-covered fibers, resin tearing, crack-tip blunting, crack deflection, shear deformation features, and crack-path reconstruction. These results indicate that, within the present two-system comparison, resin ductility-related deformation capability and local crack-tip deformability should be considered together with strength and modulus when evaluating interlaminar crack resistance. The toughening effect also showed loading-mode dependence, with Mode I improvement mainly related to crack-tip blunting and resin tearing, whereas Mode II improvement was mainly associated with matrix shear deformation, resistance to interfacial sliding, and crack-path deflection.

## 1. Introduction

Carbon fiber-reinforced polymers (CFRPs), owing to their high specific strength, high specific modulus, excellent corrosion resistance, and superior structural designability, have been widely used in aerospace, marine engineering, rail transportation, high-end equipment, and new energy structures [1,2]. With the development of advanced manufacturing and lightweight structures, composite materials are required not only to possess high static load-bearing capacity, but also to exhibit improved damage tolerance and service reliability [3,4]. For laminated composites, bonding solely by resin in the thickness direction lacks continuous reinforcement, resulting in far lower interlayer properties than in-plane properties and making the material more susceptible to damage initiation and propagation. Once delamination occurs under external loading, cracks tend to propagate along the interface, leading to a series of failure events such as further delamination, local instability, stiffness degradation, and loss of load-bearing capacity. Therefore, interlaminar fracture toughness (ILFT) is widely regarded as a key indicator for evaluating the damage tolerance and service reliability of CFRP laminates [5,6,7].

Extensive studies have been conducted worldwide to improve the ILFT of CFRP laminates. Existing approaches have mainly focused on interlaminar structural modification and matrix/interlayer toughening, such as introducing interleaves, films, nanofibrous veils, nanofillers, or hybrid toughening layers to promote crack deflection, fiber bridging, matrix deformation, and crack-path reconfiguration. Earlier representative studies have shown that electrospun nanofibrous veils, multiscale fiber-toughening strategies, interlocking thin-ply structures, and soluble or insoluble veils can improve interlaminar crack resistance by altering crack propagation paths and increasing energy dissipation [8,9,10,11]. More recent studies have further extended these strategies. Ou et al. [12] enhanced the Mode I and Mode II ILFT of unidirectional CFRP laminates by tailoring the microstructural heterogeneity of a CNT/epoxy toughening layer, showing that crack-path regulation and toughening-layer architecture strongly affect delamination resistance. Wang et al. [13] introduced polyethersulfone/graphene oxide (PES/GO) composite films into CFRP laminates and evaluated the improvement in fracture toughness using both DCB and ENF tests, with SEM observations used to analyze the corresponding damage mechanisms. Narongdej et al. [14] investigated non-woven polyamide veils under different curing pressures and showed that interlayer configuration and processing conditions can significantly influence both Mode I and Mode II ILFT. Wu et al. [15] proposed an intrinsic–extrinsic multiscale interlaminar toughening strategy using multi-walled carbon nanotubes and core–shell rubber, further demonstrating that matrix deformation, crack-path modification, and multiscale energy-dissipation mechanisms are closely related to ILFT improvement. These studies indicate that current research has increasingly focused on interlayer design, matrix modification, and crack-path control. Nevertheless, most of these approaches introduce additional toughening phases or interlaminar structures, making it difficult to isolate the role of the resin system itself under comparable fiber type, resin content, and laminate configuration.

However, in addition to externally introduced toughening phases and interlaminar structural design, the intrinsic mechanical properties of the resin matrix itself are also fundamental factors affecting ILFT. The resin matrix is not merely a simple bonding phase for fibers; its molecular chain flexibility, crosslinking structure, modulus, ductility, and deformation-related energy-dissipation capability may influence local crack-tip deformation, matrix yielding behavior, crack-tip blunting, and crack growth resistance [16]. Studies on epoxy toughening have shown that rubber particles, thermoplastic components, inorganic nanoparticles, and carbon nanomaterials can all enhance the intrinsic fracture toughness of the resin by inducing mechanisms such as shear yielding, cavitation, crack deflection, and local plastic deformation [17,18,19,20,21]. These findings suggest that improving matrix toughness not only alters the fracture behavior of the resin itself, but may also further affect crack initiation, crack propagation paths, and fracture energy dissipation in CFRP laminates. Therefore, compared with focusing solely on the role of externally added toughening phases, investigating the effect of the intrinsic properties of the resin matrix on interlaminar fracture behavior, without introducing additional interlaminar toughening structures, is helpful for achieving a clearer understanding of the role of matrix-related factors in interlaminar toughening.

In recent years, some studies have begun to focus on the transfer relationship between resin matrix toughening and interlaminar toughening in composites. Ouyang et al. [22] investigated the mechanical response of carbon fiber composites from the perspective of matrix resin toughening and pointed out that improved matrix toughness can influence internal damage evolution and residual load-bearing capacity. Wang et al. [23] toughened carbon fiber/epoxy laminates with short fibers and found that the short fibers could induce more complex crack propagation paths in the interlaminar region, thereby increasing resistance to interlaminar crack growth through mechanisms such as bridging, pull-out, and interfacial friction. Weng et al. [24] further proposed the concept of “from matrix toughening to interlaminar toughening”. After introducing short carbon nanotubes into the epoxy matrix, the maximum strain, maximum stress, and fracture toughness of the resin matrix were improved; when further fabricated into CFRP laminates, the Mode I ILFT was also markedly enhanced. These studies indicate that the deformation and energy-dissipation capability at the resin-matrix scale can be transferred through the crack-tip process zone to the interlaminar crack growth process in laminates, thereby altering fracture surface morphology and crack propagation behavior.

Although previous studies have demonstrated that externally introduced toughening phases, interfacial modification, and interlaminar structural design can effectively improve ILFT, these approaches usually alter the resin matrix, interfacial morphology, interlaminar structure, and local stress-transfer paths simultaneously. As a result, it is difficult to isolate the role of the intrinsic mechanical response of the resin matrix in the interlaminar fracture process. For engineering CFRP laminates, even when the same carbon fibers and similar laminate configurations are used, differences in strength, modulus, ductility, and fracture energy dissipation among resin systems may still significantly affect apparent interfacial fracture behavior, crack-tip process zone, and crack propagation paths. At present, comparative studies on the relationship among intrinsic resin properties, crack propagation behavior, and ILFT still need further development, particularly those aimed at evaluating the intrinsic role of the resin matrix while minimizing differences in fiber type and laminate structure. More importantly, the connection between resin properties and ILFT should be understood through the crack-tip deformation process and the corresponding loading mode. Under Mode I opening loading, resin ductility-related deformation may contribute to crack-tip blunting, resin tearing, and the formation of a more tortuous crack path. Under Mode II shear loading, the resin response may be more closely associated with matrix shear deformation, interfacial sliding resistance, crack deflection, and possible frictional dissipation. Therefore, clarifying how resin deformation capability is reflected in Mode I and Mode II interlaminar crack propagation is essential for connecting resin-level properties with laminate-level fracture resistance.

Based on the above considerations, the present work selected two representative epoxy resin systems with contrasting mechanical-performance characteristics. Group B was selected as a high-strength/high-modulus resin system, whereas Group T was selected as a high-toughness resin system with higher ductility-related deformation capability. This material selection was intended to provide a controlled comparison between a stiffness/strength-oriented resin and a toughness/deformation-oriented resin. To minimize the influence of non-resin-related variables, the same carbon fiber, resin content, calculated fiber volume fraction, unidirectional lay-up configuration, and laminate fabrication route were used for both systems. Tensile and flexural tests of neat resin castings, Mode I double cantilever beam (DCB) tests, Mode II end-notched flexure (ENF) tests, and SEM fractographic observations were conducted to examine how resin deformation capability is associated with crack-tip deformation, fracture-surface morphology, and loading-mode-dependent interlaminar fracture toughness (ILFT). The novelty of this work lies in establishing a controlled two-resin comparative framework that links neat resin mechanical response, microscopic fracture features, and Mode I/Mode II delamination resistance, rather than focusing only on external interlayers, nanofillers, or additional toughening phases.

## 2. Experiment

### 2.1. Materials

ZA55X-T800-grade carbon fiber supplied by Changsheng (Langfang) Technology Co., Ltd. (Langfang, China) was used as the reinforcing material in this study. This material was a unidirectional carbon fiber with a nominal fiber diameter of 5 μm, a tensile strength of 5880 MPa, a tensile modulus of 294 GPa, and a density of 1.80 g/cm^3^. Two epoxy resin systems with different performance characteristics were selected: a high-strength/high-modulus (HSHM) resin system (designated as Group B) and a high-toughness (HT) resin system (designated as Group T). CF/EP prepregs based on these two resin systems were prepared by a hot-melt prepregging process.

### 2.2. Preparation of Resin Casting Specimens

**Figure 1:**
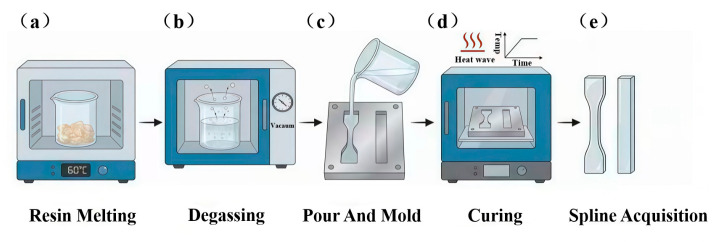
Preparation procedure of neat epoxy resin cast specimens: (**a**) resin melting; (**b**) vacuum degassing; (**c**) pouring into the mold; (**d**) curing; and (**e**) specimen acquisition.

To evaluate the mechanical properties of the two resin matrices, neat resin cast specimens were prepared for tensile and flexural tests. As shown in Figure 1a, the epoxy resin blocks were first placed in an oven at 60 °C for 2 h to ensure complete melting. The melted resin was then transferred to a vacuum oven for degassing to remove bubbles as much as possible during specimen preparation (Figure 1b). After degassing, the resin was slowly poured into a preheated metal mold coated with a release agent (Figure 1c) and then cured according to the designed curing schedule (Figure 1d). The curing schedule was 80 °C/30 min + 135 °C/60 min for Group B and 80 °C/30 min + 130 °C/30 min + 170 °C/60 min for Group T. After curing, the specimens were demolded and their edges were polished for subsequent tensile and flexural testing (Figure 1e).

### 2.3. Preparation of CF/EP Laminates

**Figure 2:**
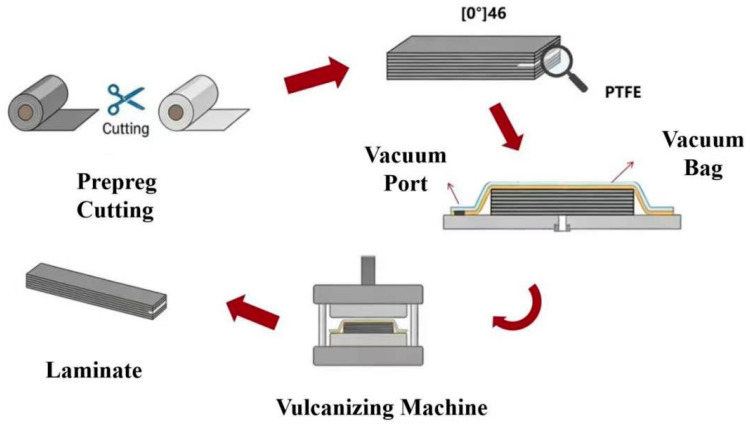
Fabrication process of composite specimens for ILFT testing.

Figure 2 illustrates the fabrication process of the composite laminates. First, the unidirectional CF/EP prepregs of the two resin systems (prepared in-house) were cut to the required dimensions and laid up unidirectionally in a [0°]_46_ sequence. To introduce an initial crack, a polytetrafluoroethylene (PTFE) film was inserted between the 23rd and 24th plies. After lay-up, the laminate was vacuum-bagged and cured. Group B was cured using a two-step schedule of 80 °C/30 min + 135 °C/60 min, whereas Group T was cured using a staged schedule of 80 °C/30 min + 130 °C/30 min + 170 °C/60 min. After curing, standard specimens for ILFT testing were obtained by machining.

The fiber volume fraction of the laminates was controlled through the prepreg design. During the hot-melt prepregging process, the areal weight of the resin film was adjusted, and the designed fiber volume fraction was calculated based on the resin content, resin density, fiber content, and fiber density. Both laminate groups were fabricated using the same prepregging and hot-press molding route to reduce processing-induced differences. No obvious dry fiber regions or severe impregnation defects were observed during specimen preparation and fracture-surface examination. It should be noted that quantitative void-content measurement was not independently performed in this study.

(1) $$V_{f}=\frac{W_{f}/\rho_{f}}{W_{f}/\rho_{f}+W_{r}/\rho_{r}}$$

where V_f_ is the fiber volume fraction, W_f_ and W_r_ are the mass fractions of fiber and resin, respectively, and $\rho_{f}$ and $\rho_{r}$ are the densities of fiber and resin, respectively. Using a resin density of 1.2 g/cm^3^, a carbon fiber density of 1.8 g/cm^3^, and a resin content of 30 wt.%, the calculated fiber volume fraction was approximately 60.9 vol.% for both laminate groups. Both laminates were prepared using the same lay-up configuration of 46 unidirectional plies. The calculated single-ply thickness was approximately 0.0685 mm, corresponding to a theoretical laminate thickness of approximately 3.15 mm. After hot-press curing, the final thicknesses of both laminate groups were close to 3 mm, indicating reasonably comparable laminate thicknesses.

### 2.4. Characterizations

#### 2.4.1. Tensile and Flexural Tests

Tensile and flexural tests of the neat resin cast specimens were carried out using an AI-7000-LAU10 universal testing machine (Gaotie Testing Instruments Co., Ltd., Dongguan, China). According to GB/T 2567-2021 [25], the tensile tests were conducted at room temperature. The specimens had an effective width of 10.0 mm and a thickness of 4.0 mm, with an extensometer gauge length of 50.0 mm and a testing speed of 2.0 mm/min. The tensile strength, tensile elastic modulus, and elongation at break were calculated as shown in Equations (2)–(4), respectively.

(2) $$\sigma_{t}=\frac{P}{\mathrm{bh}}$$

(3) $$E_{t\;}=\frac{L_{0}∆P}{\mathrm{bh}∆L}$$

(4) $$\varepsilon_{t}=\frac{∆L_{b}}{L_{0}}\;\times \;100\%$$

where $\sigma_{t}$ is the tensile strength (MPa); P is the maximum load (N); and b and h are the specimen width and thickness (mm), respectively. E_t_ is the tensile elastic modulus (MPa); L_0_ is the gauge length (mm); ∆P is the load increment in the initial linear region of the load–deformation curve (N); and ∆L is the corresponding deformation increment within the gauge length L_0_ (mm). εt is the elongation at break of the specimen (dimensionless); and $∆L_{b}$ is the elongation within the gauge length L_0_ at fracture (mm).

According to GB/T 2567-2021 [25], the epoxy resin specimens were prepared and tested in flexure. The specimen dimensions were 80 mm × 15 mm × 4 mm, the span length was 64 mm, and the loading rate was 2.0 mm/min. The flexural strength and flexural elastic modulus were calculated as shown in Equations (5) and (6), respectively.

(5) $$\sigma_{t}=\frac{3PL}{2bh^{2}}$$

(6) $$E_{t}=\frac{L^{3}∆P}{4bh^{3}∆S}$$

where $\sigma_{t}$ is the flexural strength (MPa); P is the maximum load (N); and b and h are the specimen width and thickness (mm), respectively. E_t_ is the flexural elastic modulus (MPa); L is the span length (mm); ∆P is the load increment in the initial linear region of the load–deflection curve (N); and ∆S is the corresponding mid-span deflection increment (mm).

#### 2.4.2. ILFT Tests

According to ASTM D5528-01 [26], Mode I ILFT was measured at room temperature using the double cantilever beam (DCB) test. The specimen dimensions were 240 mm × 21 mm × 3.4 mm. In the first stage of testing, the specimens were preloaded using a universal testing machine to generate a precrack of approximately 50 mm, so as to minimize the influence of the non-adhesive insert on the measured ILFT as much as possible, and were then unloaded. In the second stage, the specimens were reloaded at a loading rate of 1.0 mm/min, allowing the crack to propagate from 50 mm to 100 mm, after which they were unloaded. The Mode I ILFT, G_IC_, was calculated according to the modified beam theory method specified in ASTM D5528-01 [27], as shown in Equation (7):

(7) $$G_{\mathrm{IC}}\;=\;\frac{3P\delta }{2b(a+∆)}$$

where the P is the load; δ stands for the load point displacement; b represents the specimen width; and a is the delamination length. The ∆ can be determined experimentally by generating a least squares plot of the cube root of compliance. During the DCB test, the crack initiation point was determined from the first observable crack advance from the pre-crack tip, together with the deviation from the initial linear portion of the load–displacement curve. The specimen compliance, C = $\frac{\delta }{P}$, was obtained from the load–displacement response at each recorded crack length. The compliance calibration procedure was performed according to ASTM D5528-01 by plotting C^1/3^ against the measured crack length, and the crack-length correction factor was determined from the linear fitting. During the DCB tests, crack growth was monitored from both specimen edges during loading. The crack propagated from an initial delamination length of approximately 50 mm to a final length of approximately 100 mm. After testing, the specimen edges and fracture surfaces were inspected to verify the actual crack length and crack propagation path.

According to ASTM D7905 [27], the Mode II ILFT of the composite laminates was evaluated at room temperature using the end-notched flexure (ENF) test. The specimen dimensions were 240 mm × 21 mm × 3.5 mm, and the loading rate was 0.5 mm/min. The Mode II ILFT, GIIC, was calculated according to the compliance calibration method specified in ASTM D7905 [27], as shown in Equation (8):

(8) $$G_{\mathrm{IIC}}\;=\;\frac{3mP_{\max}^{2}a_{0}^{2}}{2b}$$

where m is the slope of the C-a^3^ calculated from the specimen compliance and the cube of the crack length; P_max_ is the maximum value of force on the load–displacement curve; a_0_ is the initial crack length (30 mm); and b is the specimen width. During the ENF tests, the onset of crack propagation was identified from the deviation from the initial linear portion of the load–displacement curve, together with the first observable change in compliance response. The maximum load, Pmax, was taken as the peak load recorded before a rapid load drop or unstable crack extension. When no abrupt load drop occurred, Pmax was defined as the maximum load reached during the recorded nonlinear loading stage. Therefore, the reported G_IIC_ values should be regarded as apparent Mode II interlaminar fracture toughness values determined according to the load–displacement response and ASTM D7905. For the ENF tests, the initial crack length was carefully controlled according to ASTM D7905. After testing, the final delamination region and fracture surfaces were inspected to confirm that crack propagation occurred from the intended pre-crack region.

#### 2.4.3. Statistical Analysis

All quantitative data are presented as the mean ± standard deviation. For the neat resin casting tests, six valid specimens were tested for each resin system. For the Mode I DCB and Mode II ENF tests, five valid specimens were tested for each laminate group. Statistical comparisons between Group B and Group T were performed using a two-tailed Student’s *t*-test. Differences were considered statistically significant at *p* < 0.05.

### 2.5. Fracture Surface Morphology Characterization

To elucidate the intrinsic fracture characteristics of the resins and the mechanisms of interlaminar crack propagation, the bending fracture surfaces of the cast resin specimens as well as the fracture surfaces from the DCB and ENF tests were examined by scanning electron microscopy (SEM). The observations focused on the surface roughness of the fracture surfaces, crack propagation paths, resin residue, tear ridges, local plastic deformation features, delamination steps, and particulate/debris-like residues. These microscopic morphological features were correlated with the tensile/flexural properties and ILFT results to analyze the differences in crack propagation behavior and energy dissipation mechanisms among different resin systems.

## 3. Results and Discussion

### 3.1. Tensile/Flexural Properties and Mechanism Analysis of the Resins

To compare the differences in the intrinsic mechanical properties of the two resin systems, tensile and flexural tests were conducted on the cast resin specimens of Groups B and T, respectively. For both tensile and flexural tests of the neat resin cast specimens, six valid specimens were tested for each resin system, and the results are presented as the mean ± standard deviation, as shown in Table 1. Overall, the HSHM resin in Group B exhibited slightly higher tensile strength/modulus and flexural strength/modulus than the HT resin in Group T, indicating greater stiffness and static load-bearing capacity. In contrast, the resin in Group T showed a significantly higher elongation at break than Group B, as confirmed by statistical analysis (*p* < 0.05), suggesting that it could withstand greater deformation before failure and thus possessed stronger ductility-related deformation and energy-dissipation capability.

**Figure 3:**
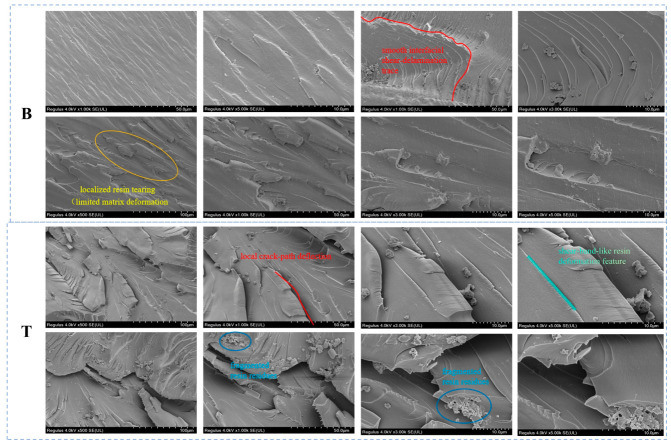
SEM morphologies of flexural fracture surfaces of resin cast specimens.

To further analyze the microscopic causes underlying the differences in flexural performance between the two resin systems, the fracture surfaces of flexural cast specimens were examined by SEM at different magnifications. As shown in Figure 3, the fracture surface of Group B was relatively smooth at low magnification, while the high-magnification SEM image further revealed cleavage-like planes and river-pattern-like features. These features suggest a relatively brittle fracture response with limited local resin deformation, rather than extensive ductile tearing. In comparison, Group T exhibited a rougher fracture surface at low magnification, and the high-magnification SEM image showed more pronounced tear-ridge-like features, shear-step-like features, and fragmented resin debris. These observations indicate that Group T underwent more obvious local deformation and energy dissipation during fracture.

These SEM observations are consistent with the subsequent ILFT results. Group B showed smoother fracture surfaces and limited local resin deformation, corresponding to lower G_IC_ and G_IIC_ values of approximately 276 J/m^2^ and 530 J/m^2^, respectively. In contrast, Group T exhibited rougher fracture surfaces with tear-ridge-like and shear-step-like features, indicating more pronounced local deformation and energy dissipation, which is consistent with its higher G_IC_ and G_IIC_ values of approximately 485 J/m^2^ and 708 J/m^2^, respectively. However, the neat resin fracture morphology should not be interpreted as direct evidence of a one-to-one transfer or amplification of resin fracture behavior into laminate fracture modes. Rather, together with the ILFT results and laminate fracture-surface observations, it provides qualitative support for understanding how resin deformation capability may be reflected in laminate-level crack propagation through matrix deformation, fiber/resin interaction, and crack-path evolution.

### 3.2. Mode I ILFT Results and Fracture Mechanism Analysis

**Figure 4:**
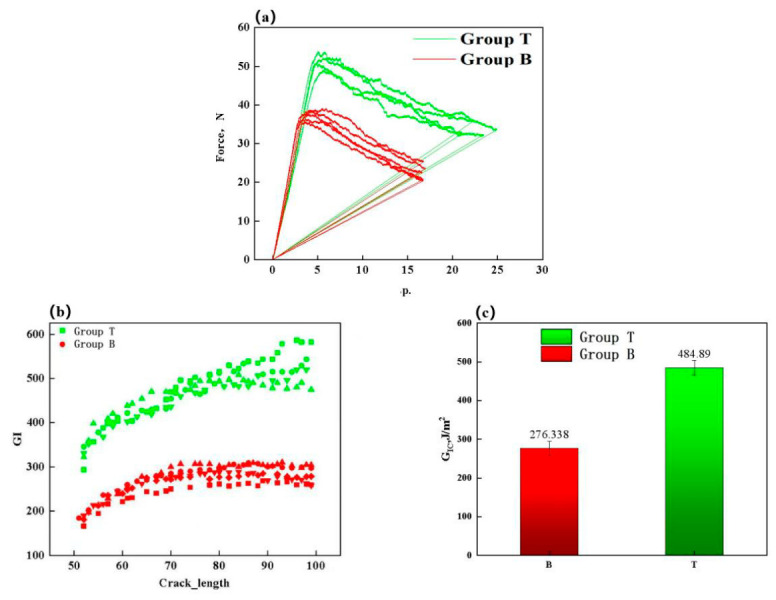
Mode I ILFT results of different laminate groups. (**a**) Load-extension curves; (**b**) R-curves; (**c**) comparison of Mode I ILFT between Group B and Group T. The values are presented as mean ± standard deviation, n = 5.

Mode I ILFT of laminates based on the two resin systems was evaluated using the DCB test. The results are shown in Figure 4. The crack initiation point was identified according to the first visible crack advance from the pre-crack tip and the deviation from the initial linear region of the load–displacement curve, and the G_IC_ values were calculated using the compliance-corrected modified beam theory method according to ASTM D5528-01. As can be seen from the load–displacement curves, the two resin systems exhibited markedly different response characteristics during crack initiation and propagation. For Group B specimens, the load dropped rapidly after reaching the peak load, with a relatively fast post-peak decay. In contrast, Group T specimens not only showed a higher peak load, but also a much more gradual post-peak decrease, maintaining a relatively high load-bearing capacity over a larger displacement range. To provide a more quantitative comparison of the R-curve behavior, the crack growth resistance slope, k_R_, was calculated by linear fitting of the G_IC_-crack length data in the stable crack propagation region of 50–99 mm. The slope k_R_ reflects the increase rate of crack growth resistance during delamination propagation. The calculated k_R_ of Group B was 0.62 ± 0.41 J·m^−2^·mm^−1^, whereas that of Group T increased to 2.89 ± 1.50 J·m^−2^·mm^−1^. This result indicates that Group T exhibited a stronger increase in crack growth resistance during stable Mode I delamination propagation, which supports the more pronounced R-curve behavior observed in Figure 4b. Further combined with the energy release rate–crack length curves, it can be seen that as the crack length increased, the G_IC_ of Group B specimens entered a relatively low plateau region after only a short propagation distance, indicating that the increase in crack growth resistance was limited, the energy dissipation zone ahead of the crack tip was small, and the material lacked sufficient sustained crack-arrest capability during stable crack propagation. By comparison, Group T specimens maintained a significantly higher G_IC_ level throughout the entire crack propagation process and exhibited a more pronounced R-curve effect; that is, as the crack propagated, the crack growth resistance of the material did not decay rapidly, but instead remained at a relatively high level over a longer propagation interval. This indicates that the HT resin system was able to develop a more sufficient plastic deformation and energy dissipation zone in the crack-tip region, thereby increasing resistance to crack growth and delaying the further development of interlaminar failure. The corresponding statistical results for GIC further confirm this trend. The average GIC of Group B was approximately 278.7 ± 18.5 J/m^2^, whereas that of Group T increased to approximately 486.7 ± 18.5 J/m^2^, representing an increase of about 74.6%. Error bars representing standard deviation were added to Figure 4c. Statistical analysis confirmed that this improvement was significant (*p* < 0.001). This significant improvement demonstrates that the HT resin system can markedly enhance the resistance of the interlaminar interface to opening-mode crack propagation, making the laminate less prone to rapid delamination failure under external loading. In other words, under the same fiber reinforcement system and similar laminate architecture, improving the toughness of the resin matrix directly enhances the interlaminar fracture behavior.

**Figure 5:**
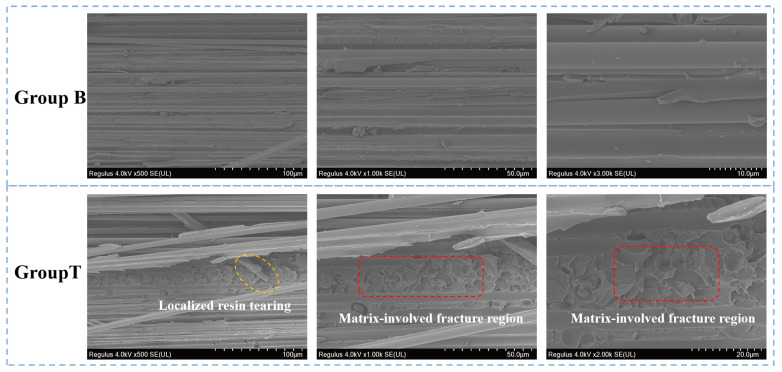
SEM images of DCB fracture surfaces for Group B and Group T, showing exposed fiber surfaces, resin residue, resin tearing, and stepped fracture features.

Further qualitative support can be obtained from SEM observations of the DCB fracture surfaces. As shown in Figure 5, the overall DCB fracture surface of Group B is relatively flat, with smooth fiber surfaces and little resin residue, indicating that the crack propagated mainly along the interlaminar interface, while the matrix played only a limited role in deformation and energy dissipation. This exhibits the features consistent with interface-dominated debonding. In contrast, the fracture surface roughness of Group T is markedly higher. The fiber surfaces are more resin residue was observed, and local features such as resin tearing, stepped fracture, and discontinuous residue can be observed, indicating that the resin matrix appeared to participate more actively in deformation and energy dissipation during crack propagation. This shows that, in Group T, the proportion of matrix-involved failure during crack growth appeared to increase, accompanied by more resin tearing and rough fracture features. Owing to the involvement of matrix plastic failure, the actual crack propagation surface area and roughness appeared rougher, which is also the qualitative morphological evidence for the sharp increase in Mode I ILFT of the laminates in Group T.

**Figure 6:**
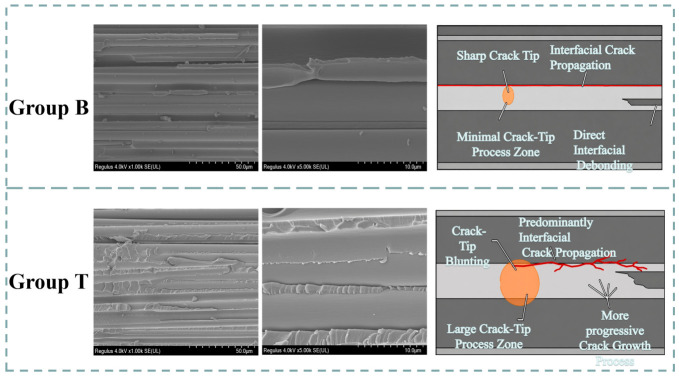
Schematic illustration of Mode I crack propagation behavior in Group B and Group T, showing interfacial delamination with different degrees of matrix participation.

To further clarify the difference in crack propagation behavior between the two resin systems during Mode I interlaminar fracture, an experimentally supported schematic illustration is proposed in Figure 6. This schematic is derived from the combined evidence of the DCB load–displacement curves, R-curve behavior, G_IC_ results and SEM fracture-surface observations, rather than from direct in situ observation of crack-tip evolution. For the high-strength, high-modulus resin system in Group B, the crack path is relatively straight, the crack tip remains relatively sharp, and the crack-tip process zone is small. This indicates that the local deformation capability of the Group B resin at the crack tip is limited, making it difficult to effectively blunt the crack tip through plastic deformation. As a result, the crack can continue to propagate along the interlaminar interface under relatively low resistance. Therefore, the Mode I interlaminar fracture process of Group B is closer to a low-energy-consumption, interface-dominated separation mode.

By contrast, the HT resin system in Group T exhibited a distinctly different crack propagation behavior. As shown in Figure 6, crack-tip blunting suggest in Group T, the crack-tip process zone may have developed, and the crack path gradually changed from relatively straight to tortuous propagation, accompanied by local deflection and progressive crack growth. This indicates that the HT resin can form a larger local energy-dissipation zone ahead of the crack tip, so that crack propagation no longer advances rapidly along a single interface, but instead proceeds through resin deformation, crack deflection, and path reconstruction. Therefore, the improvement in Mode I ILFT in Group T does not simply arise from an increase in interfacial strength, but rather from the enhanced local deformation and energy-dissipation capacity at the crack tip after resin toughening. In other words, through mechanisms such as crack-tip blunting, crack deflection, and progressive propagation, the HT resin system is associated with the intrinsic energy-dissipation capability of the resin into the interlaminar crack growth process, thereby significantly increasing G_IC_.

Accordingly, under Mode I opening loading, the translation of the resin’s intrinsic mechanical properties into ILFT is mainly reflected in the following aspects: a higher elongation at break endows the resin matrix with stronger local deformation capability at the crack tip, causing the crack tip to transition from a sharp, rapidly propagating state to a blunted, progressively propagating state; meanwhile, resin tearing and crack deflection increase the actual crack propagation path and the newly created fracture surface area, thereby converting the resin’s plastic energy-dissipation capacity into a higher G_IC_. This suggests that, within the present two-system comparison, the higher elongation at break and stronger local deformation capability of Group T are more closely associated with the improvement in Mode I interlaminar crack resistance than strength or modulus alone. However, this conclusion should not be generalized to all epoxy resin systems without further validation using a broader range of resin matrices.

### 3.3. Mode II ILFT Results and Fracture Mechanism Analysis

**Figure 7:**
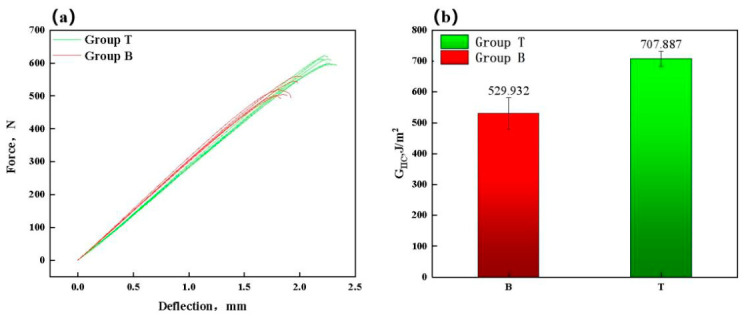
Mode II ILFT results of different laminate groups. (**a**) Load-extension curves; (**b**) comparison of Mode II ILFT between Group B and Group T. The values are presented as mean ± standard deviation, n = 5.

ENF tests were further carried out in this study to evaluate the Mode II ILFT of laminates based on the two resin systems. The results are shown in Figure 7. In the ENF load–displacement curves, the initial linear region was used to characterize the elastic response before obvious crack propagation. The onset of crack propagation was determined from the deviation from this initial linear response and the corresponding change in compliance behavior. The peak load before rapid load drop or unstable crack extension was taken as P_max_ for G_IIC_ calculation; when no abrupt load drop was observed, the maximum load reached in the nonlinear loading stage was used as P_max_. As can be seen from the load–displacement curves, both resin systems exhibited a good linear response during the initial loading stage, indicating that the overall stiffness of the specimens remained relatively stable during early loading and that no significant propagation of the interlaminar pre-crack had yet occurred. The subsequent deviation from linearity was therefore interpreted as the onset of nonlinear deformation and possible crack propagation, rather than as direct visual evidence of crack growth. As the load continued to increase, the specimens in both Group B and Group T gradually entered a nonlinear response stage. However, the specimens in Group T consistently exhibited a higher load-carrying capacity, with a peak load clearly greater than that of Group B. Moreover, after reaching the peak, Group T did not show a rapid unstable drop, but instead maintained a relatively gradual descending trend. The corresponding statistical results for Mode II ILFT show that the average G_IIC_ of Group T reached approximately 707.89 J/m^2^, representing an increase of about 33.6% compared with Group B (529.93 J/m^2^). Statistical analysis confirmed that this difference was significant (*p* < 0.05).

**Figure 8:**
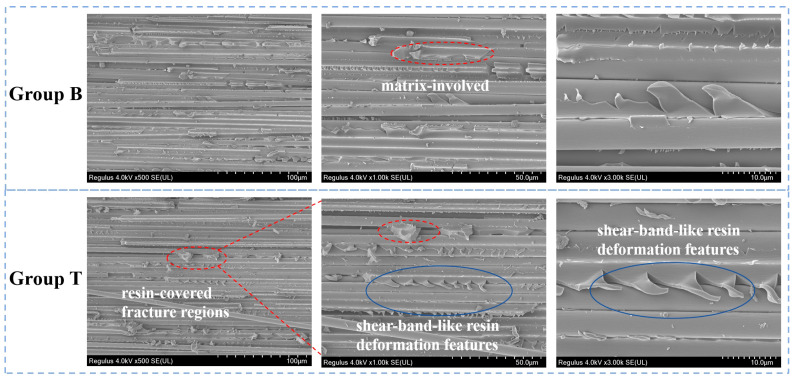
SEM images of Mode II interlaminar fracture surfaces.

SEM observations were performed on the ENF fracture surfaces of specimens from the two resin systems. As shown in Figure 8, the fracture surface of Group B was relatively smooth overall, with a more localized interfacial crack propagation path and less visible resin residue. These features suggest that the crack propagated mainly along the interlaminar interface under shear loading, with limited matrix deformation during fracture. In contrast, the fracture surface of Group T exhibited more complex morphological features, including shear-band-like resin deformation features, fragmented resin residues, microcracks, and local crack-path deflection. These features are indicated by marked regions in Figure 8 and are interpreted as morphological evidence of localized matrix shear deformation and resin tearing during Mode II crack propagation. Therefore, crack propagation in Group T was still dominated by interfacial delamination, but it was accompanied by more pronounced matrix participation and local crack-path deflection, which is consistent with its higher GIIC value.

**Figure 9:**
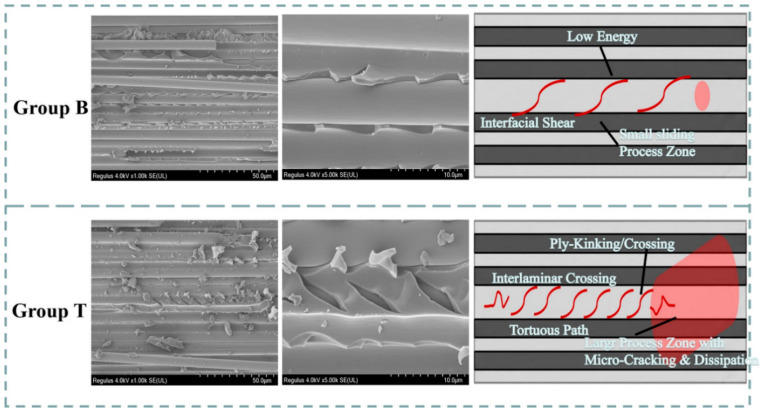
Schematic illustration of Mode II interlaminar fracture morphology and toughening mechanism.

Based on the ENF test results and fracture surface observations, Figure 9 further summarizes the crack propagation mechanisms of the two resin systems under shear loading. For Group B, the HSHM resin system, failure is dominated primarily by interfacial shear sliding. As shown in Figure 8, although the crack path in Group B exhibits some undulation under shear loading, it still propagates mainly along the interlaminar interface overall, with a relatively localized crack growth region and a small shear process zone. This indicates that the local deformation capability of the Group B resin under shear loading is limited, so the crack tends to propagate through relatively concentrated interfacial shear sliding in a more direct manner, resulting in a relatively simple energy dissipation mechanism during fracture. In contrast, the crack propagation behavior in Group T, the HT resin system, changes significantly. As shown in Figure 9, the crack path in Group T is no longer confined to a single interlaminar interface; instead, it undergoes multiple deflections under shear loading, accompanied by interlaminar crossover and local intralaminar fracture. Meanwhile, a larger shear process zone develops in the crack propagation region, indicating that shear-driven crack growth near the interface is more effectively constrained in the HT resin system and is accompanied by greater matrix shear energy dissipation. Therefore, under Mode II shear loading, the translation of the intrinsic mechanical properties of the resin into ILFT is manifested mainly as follows: the HT resin enhances resistance to interlaminar sliding through its greater shear deformability, causing crack propagation to evolve from single-interface sliding into a multi-path energy dissipation process involving shear deformation, crack deflection, interlaminar crossover, and local intralaminar failure. Unlike Mode I fracture, in which toughness enhancement is dominated by crack-tip blunting and opening-deformation energy dissipation, the improvement in Mode II fracture toughness arises more from the resin matrix’s constraint on shear sliding and its induced reconstruction of the crack path. It should be noted that the G_IIC_ obtained from the ENF test represents an apparent Mode II interlaminar fracture toughness. Under shear-dominated loading, frictional contact between the delaminated surfaces may contribute to the measured energy dissipation. Since this contribution was not independently quantified in the present study, the improvement in G_IIC_ of Group T should be interpreted as the combined effect of matrix shear deformation, crack-path reconstruction, resistance to interfacial shear crack propagation, fiber/matrix interaction, and possible frictional dissipation. It should also be noted that the resin shear properties, such as shear modulus, shear strength, and shear strain to failure, were not directly measured in this study. Therefore, a quantitative correlation between G_IIC_ and resin shear properties could not be established. The Mode II toughening mechanism proposed here is based on the ENF load–displacement behavior and post-fracture SEM morphology. Future work should include direct resin shear testing, such as Iosipescu or V-notched rail shear tests, to quantitatively clarify the relationship between matrix shear properties and Mode II ILFT. These results indicate that, for the two resin systems compared in this study, the effect of resin toughening on ILFT shows a certain dependence on loading mode, which provides useful guidance for material selection in CFRP structures subjected to different types of service loading.

### 3.4. Relationship Between Resin Deformation Capability and ILFT of Composites

To further clarify the relationship between resin properties and interlaminar fracture mechanisms, the mechanical results and SEM observations can be considered together. Group B exhibited higher strength and modulus but lower elongation at break, which was consistent with the relatively smooth fracture surfaces, smoother exposed fibers, and limited resin residue observed in the resin and CFRP fracture morphologies. These features suggest that crack propagation in Group B was more prone to occur along the resin/fiber interface, with limited matrix deformation and energy dissipation. In contrast, although Group T showed slightly lower strength and modulus, its higher elongation at break corresponded to rougher fracture surfaces, more resin-covered fibers, tear ridges, shear steps, and local crack deflection. These microscopic features indicate that the resin matrix in Group T participated more actively in the fracture process through local deformation, resin tearing, and crack-path reconstruction. Therefore, the difference in ILFT between the two laminate groups should be interpreted by combining the resin’s ductility-related deformation capability, interfacial load transfer, and crack propagation behavior, as discussed below.

**Figure 10:**
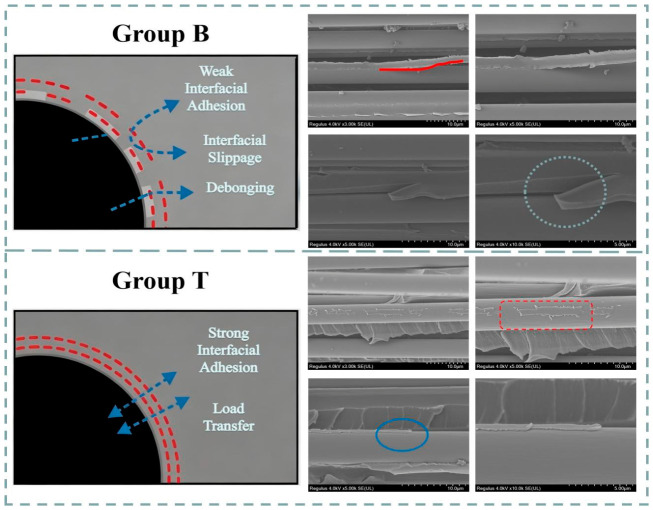
Comparison of apparent resin/carbon fiber interfacial fracture morphology in CFRP laminates with different resin systems, showing exposed fiber surfaces, resin residue, local adhesion traces, and matrix tearing features.

As shown in Figure 10, the fiber surfaces on the fracture surface of Group B are relatively smooth, with more exposed interfacial areas and less resin residue. In some local regions, features of interfacial debonding and sliding can be observed, suggesting that crack propagation in Group B was more prone to occur along the resin/fiber interface. In contrast, more resin residue, torn ridges, step-like fracture features, and local adhesion traces can be observed on the fracture surface of Group T. These features indicate that the matrix in Group T participated to a greater extent in the fracture process during crack propagation and may reflect stronger apparent fiber/resin interaction or more effective local load transfer. However, no direct interfacial characterization, such as XPS, DMA, microbond testing, or single-fiber pull-out testing, was performed in this study. Therefore, the SEM observations should be interpreted as morphological evidence of different apparent interfacial fracture behavior, rather than direct proof of improved interfacial bonding strength.

The different apparent interfacial fracture morphologies may further influence the crack propagation path and fracture mode. For Group B, the relatively lower ductility of the resin makes it difficult to form a large deformation process zone near the crack tip, and local stress concentration may not be effectively relieved. Therefore, the crack tends to propagate along the resin/fiber interface or other weak interlaminar regions, corresponding to an interface-dominated fracture mode. For Group T, the higher elongation at break and more visible resin residue suggest that the matrix can participate more actively in the fracture process. As a result, crack propagation may involve local deflection, resin tearing, microcrack formation, and partial interlaminar crossover, leading to a more tortuous apparent crack path and more complex fracture morphology.

It should be noted that fracture surface area analysis, three-dimensional surface reconstruction, and image-based quantification of mixed failure-mode fractions were not performed in this study. Therefore, the discussion of crack-path reconstruction and mixed failure modes should be regarded as a qualitative mechanistic interpretation based on post-fracture SEM morphology and ILFT results, rather than as a quantitatively verified change in fracture surface area or failure-mode proportion. Future work should include three-dimensional profilometry, image-based crack-path tortuosity analysis, or quantitative failure-mode segmentation to further verify the relationship between crack-path reconstruction and ILFT improvement. Specifically, in the DCB test, the HT resin improves Mode I ILFT mainly through crack-tip blunting, expansion of the process zone, and resin tearing, causing the crack to evolve from direct interfacial separation to tortuous and progressive propagation, thereby enhancing Mode I ILFT; in the ENF test, it improves Mode II ILFT mainly by enhancing resistance to interfacial sliding and the shear energy dissipation capacity of the matrix, thereby inducing crack deflection, interlaminar crossover, and local intralaminar failure. Thus, for the two resin systems compared in this study, the effect of the intrinsic mechanical properties of the resin on the ILFT of CFRP cannot be directly explained by any single strength or modulus parameter alone, but should instead be comprehensively analyzed in conjunction with the local deformation capability of the resin, the damage characteristics near the interface, and the evolution of the crack propagation path.

## 4. Conclusions

In this study, two epoxy resin systems with different mechanical characteristics, namely a HSHM system and a HT system, were selected to prepare unidirectional CF/EP prepregs by a hot-melt prepreg process, followed by hot-press molding into CF/EP laminates. Based on tensile/flexural tests of neat resin castings, Mode I and Mode II ILFT tests, SEM fracture observations, and schematic analysis of the toughening mechanisms, the effects of resin toughening on interlaminar fracture behavior and crack propagation were systematically investigated, and the main conclusions are as follows:

(1) Among the two resin systems, resin elongation at break and local deformation capability show a stronger correlation with CFRP ILFT than strength or modulus alone. Compared with Group B, the HT resin in Group T has slightly lower tensile strength and modulus, but its elongation at break increases from 4.0% to 6.5% (about 62.5%). Correspondingly, the Mode I ILFT increases from about 279 J/m^2^ to 487 J/m^2^ (about 74.6%), and the Mode II value increases from about 530 J/m^2^ to 708 J/m^2^ (about 33.6%). This indicates that, within the present two-system comparison, resin ductility and local deformation capability are closely associated with the improvement in interlaminar crack resistance.
(2) The fracture morphology further confirmed the different roles of the two resin matrices during delamination. Group B showed smoother fracture surfaces, more exposed fiber surfaces, and less visible resin residue, indicating a more interface-dominated crack propagation process. In contrast, Group T exhibited rougher fracture morphologies, more resin residue, resin tearing, stepped fracture features, shear-band-like resin deformation features, fragmented resin residues, and local crack-path deflection. The apparent resin coverage area fraction also increased markedly in Group T, supporting greater matrix participation during Mode I crack propagation.
(3) This study reveals a loading-mode-dependent toughening mechanism for CFRP laminates governed by resin deformation capability. Under Mode I opening loading, the improvement in ILFT is mainly associated with crack-tip blunting, resin tearing, and progressive crack propagation. Under Mode II shear loading, the improvement is more closely related to matrix shear deformation, resistance to interfacial sliding, possible frictional dissipation, and crack-path reconstruction. The contribution of this work lies in linking neat resin mechanical response, post-fracture morphology, and Mode I/Mode II ILFT within a controlled comparative framework, providing a useful reference for resin selection and toughness-oriented design of carbon fiber/epoxy prepreg systems.

## Figures and Tables

**Table 1:** Mechanical properties of different resin systems. The values are presented as mean ± standard deviation, n = 6.

Sample	Tensile Strength (MPa)	Tensile Modulus (GPa)	Flexural Strength (MPa)	Flexural Modulus (GPa)	Elongation at Break (%)
Group B	95 ± 0.94	3.47 ± 0.03	152 ± 5.3	3.40 ± 0.03	4.0 ± 0.2
Group T	92.2 ± 0.51	3.14 ± 0.05	141 ± 3.7	2.97 ± 0.06	6.5 ± 0.5

## Data Availability

The original contributions presented in this study are included in the article. Further inquiries can be directed to the corresponding authors.
